# Transcriptomics and Antioxidant Analysis of Two Chinese Chestnut (*Castanea mollissima* BL.) Varieties Provides New Insights Into the Mechanisms of Resistance to Gall Wasp *Dryocosmus kuriphilus* Infestation

**DOI:** 10.3389/fpls.2022.874434

**Published:** 2022-04-15

**Authors:** Cancan Zhu, Wu Wang, Yu Chen, Yuqiang Zhao, Shijie Zhang, Fenghou Shi, Muhammad Khalil-Ur-Rehman, Niels J. Nieuwenhuizen

**Affiliations:** ^1^Institute of Botany, Jiangsu Province and Chinese Academy of Sciences, Nanjing, China; ^2^College of Forestry, Nanjing Forestry University, Nanjing, China; ^3^Department of Horticultural Sciences, The Islamia University of Bahawalpur, Bahawalpur, Pakistan; ^4^The New Zealand Institute for Plant and Food Research Ltd., Auckland, New Zealand

**Keywords:** Chinese chestnut, *Castanea mollissima*, gall wasp, *Dryocosmus kuriphilus*, RNA-seq, POD, WGCNA, MAPK

## Abstract

Chinese chestnut is a popular fruit tree with a high nutritional value of its nuts, which can suffer from infestation by the chestnut gall wasp *Dryocosmus kuriphilus* (GWDK) that results in gall formation and resultant loss of production and profitability. The physiological and molecular mechanisms of GWDK resistance found in certain genotypes currently remains elusive. To gain new insights into this phenomenon, a series of RNA-Seq integrated with metabolomic profiling experiments were executed to investigate the chemical and transcriptional differences in response to GWDK infestation in two contrasting chestnut varieties grown in China (the susceptible “HongLi,” HL and the partially resistant “Shuhe_Wuyingli,” SW). Three time points were selected for comparison: The initiation stage (A), growth stage (B), and maturation stage (C). Results showed that concentrations of hydrogen peroxide (H_2_O_2_) and the activities of peroxidase (POD) and superoxide dismutase (SOD) enzyme were elevated in the resistant SW leaves compared with those in HL leaves at all three developmental stages, while catalase (CAT) and polyphenol oxidase (PPO) activities were mostly higher in HL leaves. RNA-Seq transcriptomic analyses of HL and SW leaves revealed that various metabolic pathways involved in GWDK stress responses, such as plant hormone signal transduction, MAPK signaling, and the peroxisome pathway, were enriched in the contrasting samples. Moreover, the weighted gene co-expression network analysis (WGCNA) of differentially expressed genes in the POD pathway combined with transcription factors (TFs) indicated that the expression of TF members of bHLH, WRKY, NAC, and MYB family positively correlated with POD pathway gene expression. The TFs *CmbHLH130 (EVM0032437)*, *CmWRKY31 (EVM0017000)*, *CmNAC50 (EVM0000033)*, and *CmPHL12 (EVM0007330)* were identified as putative TFs that participate in the regulation of insect-induced plant enzyme activities in chestnut, which may contribute to GWDK resistance in SW. Expression levels of 8 random differentially expressed genes (DEGs) were furthermore selected to perform quantitative reverse transcription PCR (qRT-PCR) to validate the accuracy of the RNA-Seq-derived expression patterns. This study guides the functional analyses of further candidate genes and mechanisms important for GWDK resistance in chestnuts in the future as well as can help in identifying the master transcriptional regulators and important enzyme steps that support major insect defense pathways in chestnut.

## Introduction

*Castanea mollisima* Blume, also known as Chinese chestnut, belongs to the Fagaceae family and is an economically valuable tree in China. More than 300 wild and cultivated varieties have been reported (Nishio et al., 2021). Most of Chinese chestnut species were reported as highly resistant to abiotic stresses while being susceptible to herbivory infestations (Acquadro et al., 2020); specialized metabolites and antioxidants have been reported to modulate interactions of plants with their abiotic environment (Chapman et al., 2019). The Asian chestnut gall wasp, *Dryocosmus kuriphilus* Yasumatu (Hymenoptera: Cynipidae), is one of the main pests of the genus *Castanea* and can form a large number of galls on plant leaves or buds. *D. kuriphilus* can also harm the chestnut, cause the leaves to be deformed, and may result in the death of whole branches. As a result from the infestation, the fruiting branches and the vegetative branches cannot grow normally, which eventually leads to a progressive loss of the photosynthetic biomass (Zhu et al., 2019). This pest is also considered as a significant problem in other di- and monocot species such as in grape (Granett et al., 2001), wheat (Smiley et al., 2004), blueberry (Sampson et al., 2002), and rice (Way et al., 1991). *D. kuriphilus* infestation in chestnut is difficult to prevent and control because most of its life cycle occurs inside the galls.

Chestnut galls induced by *D. kuriphilus* are among the most fascinating structures found in fruit crops, as they are the product of sophisticated plant-insect interactions (Ronquist and Liljeblad, 2001; Stone and Schönrogge, 2003). Insect-induced galls are an extreme example of an extended phenotype (Martinson et al., 2021), in which the galls are produced and maintained by plant genes and enzymes, while the initiation, development, and morphology of the galls are rather controlled by the insect through manipulation of the cells of the plant (Raman, 2011; Korgaonkar et al., 2021). To date, the genes/pathways of plants that respond to *D. kuriphilus* infestations and that affect the galls productions need to be further clarified.

External factors that affect the chestnut galls productions include the phenology of the host, the phenology of the parasitoids, and environmental conditions such as temperature. Apart from these factors (Geng et al., 2015), the genetics of the plant host may also play an important role and affects the *D. kuriphilus* feeding. In previous studies, a chestnut variety “Shuhe_Wuyingli,” SW, was identified, which is partially resistant to gall infestations (Geng et al., 2015; Zhu et al., 2019). Morphological and cytochemical differences between susceptible and resistant varieties have been surveyed to clarify the nature of the resistance on the chestnut trees to the gall wasps (Matsui and Torikata, 1970). In this study, the GWDK-susceptible cultivar of “HongLi,” HL, and GWDK-partially resistant “SW” chestnut were used to further explore the molecular mechanism of GWDK-resistance through physiological and temporal RNA-Seq analysis.

Plants have been interacting with herbivores for millions of years and evolved sophisticated strategies to protect themselves against attacks by biotic stressors. Previous studies have demonstrated that herbivory feeding commonly induces jasmonic acid (JA)-dependent defense pathways in tea (Jing et al., 2021), soybean (Selig et al., 2016), and tomato (Bosch et al., 2014), where jasmonate can contribute to protecting plants against herbivores. The induction of JA in plants that experience insect damage can lead to increased production of secondary defense metabolites such as terpene compounds or increase the polyphenol oxidase (PPO) activity to maintaining considerable resistance against insect pests through a variety of possible mechanisms. Terpenes are important compounds involved in the interactions between plants and insects as well between plants and pathogens, such as in tea leaves, where the homoterpene *(E)*-4,8-dimethyl-1,3,7-non-atriene (DMNT) induced the accumulation of JA and thus promoted resistance in neighboring uninfested plants to herbivorous insects. In this process, activation of the terpene biosynthesis pathway genes in response to the herbivory infestations is highly beneficial (Jing et al., 2021).

Another significant pest and stress defense mechanisms that has been reported is the antioxidant defense system. When plants are infested by insects, induction of antioxidant enzymes is part of the early responses to the herbivory signals. This includes antioxidant enzymes and low-molecular antioxidants, such as superoxide dismutase (SOD), peroxidase (POD), catalase (CAT), phenylalanine ammonia lyase (PAL), and hydrogen peroxide (H_2_O_2_) (War et al., 2012). SOD can convert superoxide radicals (O^–2^) into H_2_O_2_ (Liochev and Fridovich, 2007); POD and CAT play major roles in reducing/scavenging H_2_O_2_ to water using different substrates as electron donors. The rapid detoxification of both O^–2^ and H_2_O_2_ are essential processes to prevent excessive oxidative damage (Shi et al., 2014). Several studies have demonstrated that the activity of antioxidant enzymes was positive correlated with plant tolerance to biotic stresses, including pests and fungi (Chojak-Koźniewska et al., 2018).

The Chinese chestnut genome sequence was recently released (Wang et al., 2020), and some studies on the transcriptional regulation of chestnut secondary metabolites and QTL mapping of chestnut disease resistance (Barakat et al., 2012) have been completed. However, a comprehensive transcriptome analyses to identify gene expression and expressional variations of genes of the Chinese chestnut in response to GWDK infestation using comprehensive transcriptome resources are still lacking. Deep sequencing by RNA-Seq-based approaches can enable researchers to generate an unprecedented global view of the transcriptome changes and to identify the signaling pathways responsible for plant defense to various biotic stresses. Recently, several studies have used RNA-Seq assays to quantify changes in the transcriptome upon herbivory infections, and a small subset of novel defense-response candidate genes were also identified (Huang et al., 2020; Trujillo-Moya et al., 2020).

To determine the gene expression profile changes in chestnuts leaves upon gall wasp infestation and to identify developmental stage-specific genes and pathways involved in this interaction, in this study, temporal RNA-Seq was combined with antioxidant enzyme metabolomic profiling. This approach included an in-depth characterization of the genes and pathways altered in susceptible vs. partially resistant chestnut-GWKD interactions. This dataset provides new insights into the chestnut-GWKD interactions and identifies candidate genes/enzymes in plant responses to GWDK infestation that could contribute to the molecular-aided breeding of gall wasp–resistant chestnuts and other woody plants.

## Materials and Methods

### Materials and Treatments

**FIGURE 1:**
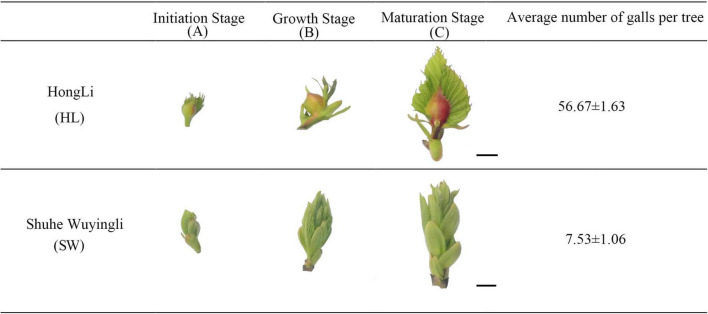
Physicochemical changes during “HongLi (HL)” and “Shuhe_Wuyingli (SW)” bud development. The average number of galls per tree were analyzed. Mean ± standard error (*n* = 12). Scale bar = 1 cm.

Four-year-old Chinese chestnut (*Castanea mollissima* Blume) plants of two varieties, namely, “HL” and “SW,” were planted in Chinese chestnut germplasm resources unit, Nanjing, Jiangsu Province, China, under same growth conditions (i.e., temperature, humidity, and light). The variety “HL” was susceptible to GWDK infestation, while “SW” was partially resistant to GWDK infestation as manifested by the appearance of less and smaller galls on the resistant plants following the methodology by Acquadro et al. (2020). Buds with galls were harvested from 12 plants at 3 time points starting from budburst from April 7 until April 26 [Initiation stage (A) at April 7, growth (B) at April 15, and maturation (C) at April 26] in 2019 (Figure 1). Each sample contained three biological replicates. Bud samples of chestnut were harvested from 12 plants (four plants per replicate) and used for the enzyme activity test, RNA extraction, and RNA-sequencing. The fresh buds were sampled and frozen in liquid nitrogen and stored at –80°C until further use.

### Determination of Hydrogen Peroxide Content

The H_2_O_2_ content (n mol g^–1^ fresh weight) from chestnut buds was assessed using the xylenol orange method, as described by Loreto and Velikova (2001). The absorbance was measured using spectrophotometer at 560 nm, and the experiment was repeat at least 3 times.

### Antioxidant Enzyme Activities Assay

All the antioxidant enzymes described below were extracted by homogenizing 0.3 g of frozen buds in phosphate buffer solution (pH 7.4 and 4% polyvinylpolypyrrolidone) and then centrifugated at 16,000 × *g* for 30 min at 4°C; the supernatants were collected and used for antioxidant enzyme activities assays. The activities of SOD, CAT, PPO, and POD were assayed according to the methodologies proposed by Beers and Sizer (1952), Giannopolitis and Ries (1977), Wissemann and Lee (1980) and Amako et al. (1994), respectively, with some modifications. The activities of CAT, POD, PPO, and SOD were determined from the measurement of absorbance at 240, 420, 410, and 560 nm, respectively. The results of aforementioned enzymes were expressed as U mg^–1^ FW; three biological replicates were utilized to make each of the measurements.

### RNA Preparation, Transcriptome Sequencing, and Functional Annotation

A total of 100 mg frozen bud samples for 2 species of 3 development stages were ground in a mortar under liquid nitrogen, and the resultant powder was used for RNA extraction with the TRIzol^®^ extraction reagent (Invitrogen, United States.). Nano Drop and Agilent 2100 bioanalyzer (Agilent Technologies, United States) were used to assess the quality of the RNA sample, and high-quality RNA samples with an A260/A280 ratio of 1.8–2.1 were used as template material for RNA-Seq and quantitative reverse transcription PCR (qRT-PCR) analyses. In total, 18 libraries were prepared (i.e., 3 biological replicates per sample) and sequenced using the SE 50 using Illumina Hiseq2500 platform (BGI, Shenzhen, China); the raw reads were filtered by removal of adapter and low-quality sequences using SOAPnuke v1.5.2. Data are available under NCBI SRA accession PRJNA791965. The clean reads were mapped to Chinese chestnut genome^1^ using HISAT2 (v2.0.4); fragments per kilobase per million reads (FPKM) values were applied to calculate the gene expression levels. The DESeq (V1.4.5) package was used to identify the differentially expressed genes (DEGs); false discovery rate (FDR) value ≤ 0.05 and |log2Fold Change| ≥ 1 were set for significant gene expression differences between two samples. To gain insights into the changes in phenotype, the enrichment analysis of the DEGs was performed with gene ontology (GO)^2^ and Kyoto Encyclopedia of Genes and Genomes (KEGG^3^) annotation. Annotations of some DEGs also referred to the genome of hardwood^4^. The significant levels of the terms and pathways were corrected using a rigorous threshold (*q*-Value ≤ 0.05) *via* Bonferroni correction.

### Candidate Differentially Expressed Genes Validation by Quantitative Reverse Transcription-PCR

Expression pattern verification of 8 randomly selected DEGs from RNA-Seq was performed using qRT-PCR, as described previously (Zhu et al., 2019). Complementary DNA (cDNA) was transcribed using the Takara Prime Script TM-RT PCR reagent Kit (Takara, Japan) according to the manufacturer’s instructions. Specific primers (Supplementary Table 1) for qRT-PCR were designed using the Primer Premier 5 software. *Actin* gene was selected as internal reference in this study. qRT-PCR was conducted using the ABI7500 RT-PCR system according to the manufacturer’s instructions (Applied Biosystems, Foster City, CA, United States). All reactions and non-template controls were performed in triplicate. Relative transcription levels were calculated using the 2^–ΔΔ*Ct*^ method (Livak and Schmittgen, 2001). Data are expressed as mean ± standard error (SE) (*n* = 3).

### Correlation Network Construction and Visualization

Candidate genes associated with the GWDK infestation were identified based on the correlation of transcript levels with antioxidant enzymes activities during berry development among the 2 varieties. Weighted gene coexpression network analysis (WGCNA) was conducted to get the gene coexpression network based on total 29,886 DEGs, Visualization of the gene coexpression network was performed using Cytoscape (version 3.8.2) (Vasaikar et al., 2018). Correlation network was performed using the OmicStudio tools^5^.

### Statistical Analysis

Figures were made using GraphPad prism 8.0 (San Diego, CA, United States.). All the analyzed data were expressed as means ± standard error (SE). SPSS Version 17.0 (SPSS Inc. Chicago, IL, U.S.A.) and Excel software were used to perform statistical analyses. A one-way ANOVA with a Duncan’s *t*-test was used to evaluate the significant differences (*p* ≤ 0.05). TBtools (v 0.665) software were applied for heatmaps.

## Results

### Basic Physicochemical Parameters of Bud Development and Galls Formation

The basic physiological indicators of the different development stages of gall formation by *D*. *kuriphilus* are shown in Figure 1. For “HL,” the galls are smaller at the initiation stage (A), while during the bud development, the galls become larger and more visible (B). At the maturation stage (C), galls in the buds showed a darker red appearance, while in the buds of “SW,” galls were more difficult to spot during all the development stages, and the average number of galls in the “HL” was significantly higher than “SW” (7.5 times higher).

### Hydrogen Peroxide Content and Antioxidant Enzyme Activities of “HongLi” and “Shuhe_Wuyingli” Buds

**FIGURE 2:**
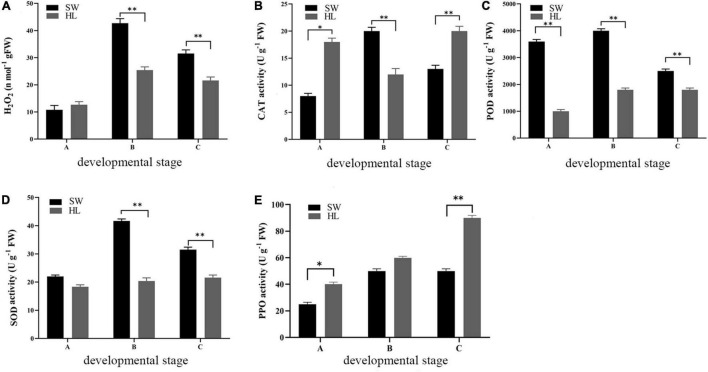
Hydrogen peroxide (H_2_O_2_) content **(E)** and antioxidant enzyme activities of “HL” and “SW” buds during infestation by gall wasp *Dryocosmus kuriphilus* (GWDK) gall wasps [**(A)** polyphenol oxidase (PPO); **(B)** catalase (CAT); **(C)** peroxidase (POD); and **(D)** superoxide dismutase (SOD)]. Asterisks indicate a significant difference (*P < 0.05, **P < 0.01) between ‘HL’ and ‘SW’.

Hydrogen peroxide content is an important indicator of oxidative damage. Therefore, the H_2_O_2_ content was investigated during the 3 development stages of “HL” and “SW” buds. At the initiation stage A, the H_2_O_2_ content in buds of the 2 experiment species was similar, while in the growth and maturation stage (B and C), increasing H_2_O_2_ was observed compared with that in the initiation stage. The maximum content of H_2_O_2_ (42.7 n mol g^–1^ FW) was observed in the “SW” buds at the growth stage (B), and the content of H_2_O_2_ was significant higher in “SW” buds at the growth and maturation stages compared with “HL” (Figure 2A).

The antioxidant enzymes system, which includes CAT and POD, PPO, and SOD, play significant roles in preventing or alleviating the herbivory damage of plants resulting from reactive oxygen. These enzyme activities were therefore measured in “HL” and “SW” buds to evaluate the relative antioxidant levels. As shown in Figures 2B–E, at the initiation stage, PPO and CAT activity was higher in “HL” buds, while in contrast, higher POD and SOD activities were observed in “SW” buds. At the growth and maturation stages (B and C), the activity of CAT, POD, and SOD was generally higher in “SW” buds compared with that in “HL.” The only exception was the CAT activity at stage C, which was higher in HL. In contrast to CAT, POD, and SOD, a higher PPO activity was observed in “HL” buds during all three stages, which was statistically significant for stages A and C. Overall, in the majority of cases, the four enzyme activities were lowest at the initially time (i.e., stage A) and increased rapidly toward stage B and remained stable or increase slightly for rest of the examination period. The higher values of H_2_O_2_ content and antioxidant enzyme activities were generally observed in the resistant “SW” buds, as compared with “HL” buds, apart from the PPO activity. The values of these parameters almost showed an increasing to decreasing trend during the development of both “HL” and “SW” buds.

### Libraries Construction and Sequencing

The samples of the two chestnut varieties at three developmental stages (i.e., initiation, growth, and maturation) were subjected to RNA-Seq transcriptomics analysis. High-throughput RNA-Seq generated between 1.19 and 1.21 Gb of clean base reads from each library (Table 1). After stringent filtering of low-quality sequences, on average of 23.87 M clean reads were obtained with quality scores > Q30, which represented > 91.12% of the reads that were subsequently mapped to the reference genome (see text footnote 1). The aligned ratios of the reads ranged from 71.01% to 74.96% among the eighteen libraries, and 64.95–71.97% of the reads were uniquely mapped (Table 1). These data represented a high sequencing depth and quality sufficient for further transcriptomics analysis.

**TABLE 1:** Statistical results of the RNA-Seq data.

Classification	Maximum	Minimum	Average
Number of clean bases (Gb)	1.21	1.19	1.2
Number of clean reads (M)	24.1	23.87	23.98
Number of reads aligned (M)	18.07	16.99	17.51
Percentage of reads aligned (%)	74.96	71.01	73.01
Number of unique mapping reads (M)	17.24	15.26	16.58
Percentage of unique mapping reads (%)	71.97	64.95	68.82
Q30 (%)	92.12	91.12	91.61

*Q30 indicates the percentage of data/bases with a phred quality score > 30. Phred quality score > 30 means > 99.9% base call accuracy.*

**FIGURE 3:**
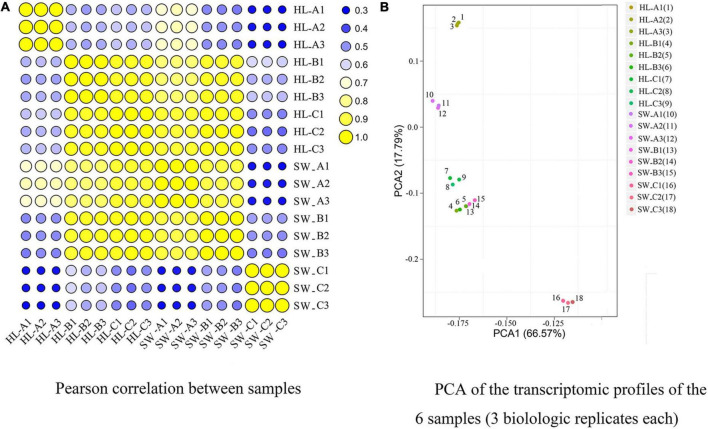
Pearson’s correlation matrix **(A)** and principle component analysis (PCA) **(B)** of differentially expressed genes (DEGs) were performed on three biological replicates of each sample sets to evaluate correlations and variance between samples. “HL” and “SW,” “HongLi” and “Shuhe_Wuyingli” cultivars, respectively; A/B/C: bud infestation stage infestation/growth/maturation.

The expression levels of all the sequenced samples were then calculated *via* the FPKM method and subjected to principle component analysis (PCA) and Pearson correlation analysis (Figure 3A). All samples showed a degree of correlation (the within-group correlation was at least R^2^ > 0.962). PCA of the expression profiles of the 18 libraries [i.e., 6 samples and 3 biological replicates (Figure 3B)] revealed that the samples from the three different stages and 2 cultivars could be clearly separated, confirming that between-stage variation was relatively high and that the three stages showed distinct global expression patterns, which were suitable for further analysis. PCA data (PCA1 variance explained 66.57% and PCA2 17.79%) represent 84.36% of the variance across the two dimensions. Overall samples HL and SW stages B clustered most closely together and both clustered closely with HL stage C, while the other combinations were more distant.

### Identification of Differentially Expressed Genes

**FIGURE 4:**
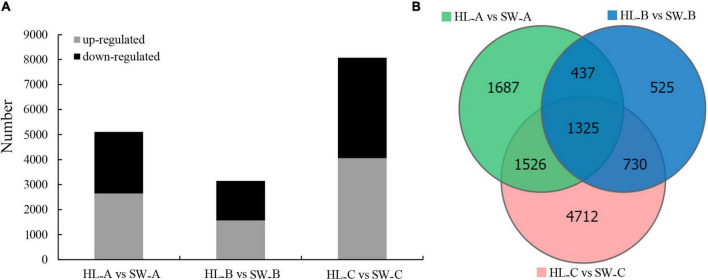
Differential gene expression in chestnut buds in the presence of the GWDK in resistant/susceptible cultivars. **(A)** The number of upregulated and downregulated DEGs (log_2_ (fold change) ≥ 1 and false discovery rate (FDR) < 0.05). **(B)** Quantity statistics Venn diagrams of differentially expressed genes among the three sampling groups (log_2_ (fold change) ≥ 1 and FDR < 0.05).

After the estimating of the gene expression levels through the FPKM method, 12,793 protein-coding DEGs were identified (log2 (fold change) ≥ 1 and FDR < 0.05) and further analyzed as the critical genes that associate with GWDK infestation (Figure 4A). Among them, 4,975, 3,017, and 8,293 DEGs were differentially expressed (log_2_ (fold change) ≥ 1 and FDR < 0.05) at stages A, B, and C, respectively, when comparing the two cultivars (Figure 4B).

### Gene Ontology and Kyoto Encyclopedia of Genes and Genomes Functional Enrichment Analysis of Differentially Expressed Genes

**FIGURE 5:**
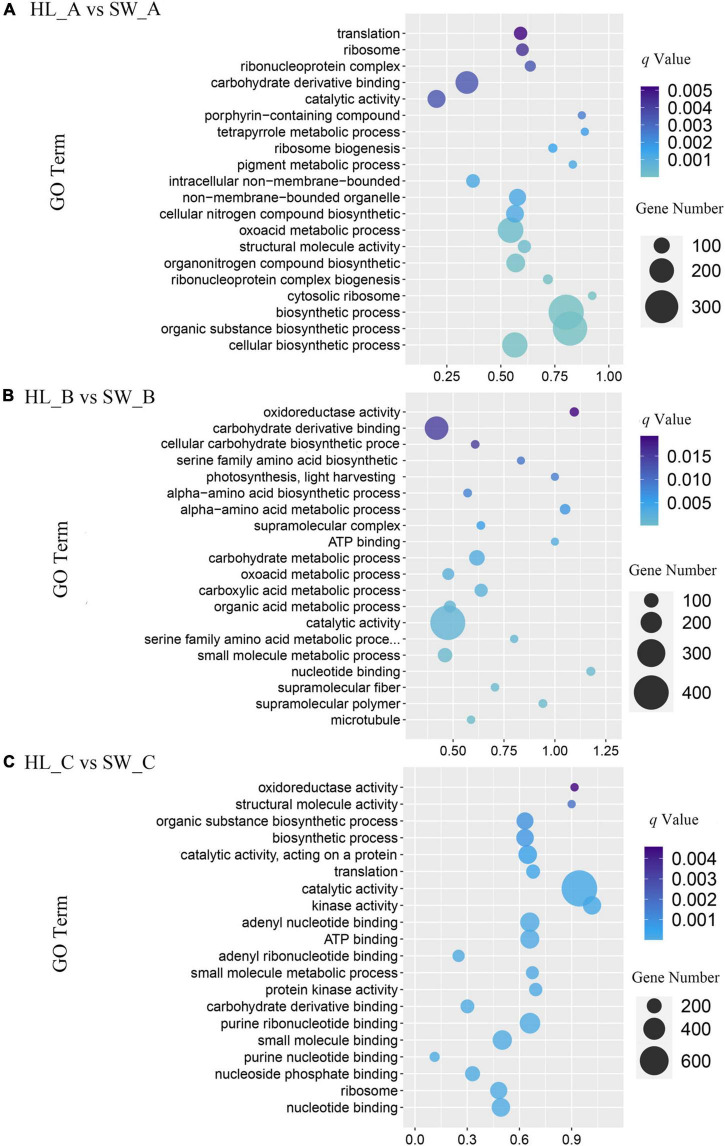
Gene ontology (GO) pathway analysis of differentially expressed genes. Advanced bubble chart shows enrichment of differentially expressed genes in certain pathways. **(A)** HL_A vs. SW_A; **(B)** HL_B vs. SW_B; and **(C)** HL_C vs. SW_C. Count represents the DEGs number, and gene ratios are calculated as the ratio of DEGs in each GO pathway compared with total number of DEGs.

To further investigate the functions of the detected 12,793 DEGs [log_2_ (fold change) ≥ 1], GO-based enrichment was performed with a threshold value of *p*-Value < 0.05 to evaluate significantly enriched GO pathways. A total of 6,674 DEGs (52.16%) were annotated as “biological process,” which contained the majority of GO terms, followed by 4,820 DEGs (37.15%) annotated as “cellular component” and 5,162 DEGs (39.79%) that were annotated as “molecular function.” Through GO assignments, the assigned DEGs were divided into 41 functional groups. The major subcategories along with the analysis of all the transcripts among the three different stages are shown in Figure 5A. Transcripts associated with small molecule binding (GO: 0044283) accounted for the highest number of enriched GO terms among stage A comparisons, whereas catalytic activity (GO: 0003824) include the highest number of DEGs among both stages B and C classification (Figures 5B,C).

Various DEGs are coordinately expressed to perform their different biological functions; therefore, the KEGG pathway-based analysis of DEGs was performed to identify the candidate pathways and genes associated with GWDK infestation. Based on the KEGG analysis, 12,793 DEGs were allocated to 136 pathways with the most enriched pathways as shown in Table 2 (*q*-Value < 0.05, top 10 numbers of DEGs). In the comparisons of stages A, B, and C, plant hormone signal transduction (ko04075) and MAPK signaling pathway (ko04016) were detected as the top 2 significant KEGG pathways (*q*-Value < 0.0053) at all three stages and included the highest number of DEGs. Furthermore, in the comparison at stages B and C, the peroxisome pathway (ko04146) was also significantly enriched (*q*-Value of 0.049 and 1.28E-05, respectively). GO and KEGG annotations for all the DEGs are shown in Supplementary Table 2.

**TABLE 2:** Significantly enriched pathways of differentially expressed genes (DEGs) among HL and SW samples through KEGG enrichment analyses at bud infestation stages A, B, and C.

Pathway ID	Pathway	Number of annotated	Up-regulated	Down-regulated	*q*-Value (<0.05)
			
		log2 (fold-change) ≥ 1			
**HL_A vs SW_A**					
ko04075	Plant hormone signal transduction	120	79	41	8.65E-06
ko04016	MAPK signaling pathway	105	73	32	1.67E-02
ko03010	Ribosome	37	17	20	3.25E-03
ko03008	Ribosome biogenesis in eukaryotes	25	12	13	2.09E-05
ko04145	Phagosome	10	8	2	4.96E-02
ko00196	Photosynthesis - antenna proteins	9	9	0	4.92E-06
ko03050	Proteasome	8	1	7	2.45E-02
ko00590	Arachidonic acid metabolism	4	3	1	3.49E-02
ko00860	Porphyrin and chlorophyll metabolism	3	3	0	4.48E-06
ko00750	Vitamin B6 metabolism	3	1	2	4.56E-04
**HL_B vs SW_B**					
ko04016	MAPK signaling pathway	55	28	27	0.005288
ko04075	Plant hormone signal transduction	36	22	14	0.019426
ko00511	Other glycan degradation	11	9	2	0.017392
ko03008	Ribosome biogenesis in eukaryotes	10	5	5	0.049904
ko04146	Peroxisome	6	4	2	0.049904
ko01212	Fatty acid metabolism	5	4	1	0.025405
ko01200	Carbon metabolism	5	1	4	0.017667
ko00051	Fructose and mannose metabolism	5	0	5	0.00222
ko00943	Isoflavonoid biosynthesis	4	2	2	0.005288
ko00860	Porphyrin and chlorophyll metabolism	3	2	1	0.000547
**HL_C vs SW_C**					
ko04075	Plant hormone signal transduction	187	65	122	3.93E-08
ko04016	MAPK signaling pathway	179	104	75	2.70E-08
ko00511	Other glycan degradation	30	8	22	1.91E-02
ko03050	Proteasome	18	4	14	4.62E-06
ko00941	Flavonoid biosynthesis	17	11	6	5.48E-03
ko04146	Peroxisome	16	8	8	1.28E-05
ko00564	Glycerophospholipid metabolism	16	6	10	4.19E-04
ko00592	alpha-Linolenic acid metabolism	14	11	3	4.19E-04
ko00562	Inositol phosphate metabolism	14	5	9	7.40E-03
ko01212	Fatty acid metabolism	13	5	8	8.94E-03

### Transcripts Associated With the Plant Hormone Signal Transduction and MAPK Signaling Pathway

In this study, 157 and 178 transcripts (log_2_ (fold change) ≥ 1 and *q*-Value < 0.05) linked to plant hormone signal transduction (Supplementary Table 3) and MAPK signaling (Supplementary Table 4) were identified by comparing the three development stages of two Chinese chestnut species, respectively. In the hormone signal transduction pathway, the ABA, auxin, brassinosteroid, cytokinin, ethylene, gibberellin, and jasmonic acid (JA) signaling pathways were further analyzed (Supplementary Table 3). Among them, the JA signaling pathways genes were commonly reported and are often associated with plant herbivory defense. In this pathway, 19 DEGs were identified, which were annotated as coronatine-insensitive, TIFY or MYC, and bHLH transcription factors. Across the three stage comparisons, 5 TIFY protein (i.e., *EVM0015448, EVM0017479, EVM0018121, EVM0013408*, and *EVM0017717*) were all higher expressed in SW compared with HL (Supplementary Table 3). In the MAPK signaling pathway, the majority of DEGs were annotated as leucine-rich repeat (LRR) receptor-like kinase/receptor like (85 out of 178) and members of the transcription factor family WRKY (17 out of 178) and bHLH [9 out of 178 (Supplementary Table 4)]. Among the 178 DEGs of the MAPK signaling pathway, *EVM0018807* and *EVM0025267* were detected as the top two highest most highly expressed genes at the maturity stages A and B, respectively, which were annotated as acanthoscurrin-1-like and DNA damage-repair/toleration protein DRT100-like, respectively. *EVM0025267* showed consistently higher expression in the HL variety at all three stages, while *EVM0018807* was most downregulated at stage B in the HL (33-fold) variety vs. most downregulated at stage C in SW (350-fold).

### Transcription Factor Analyses

**FIGURE 6:**
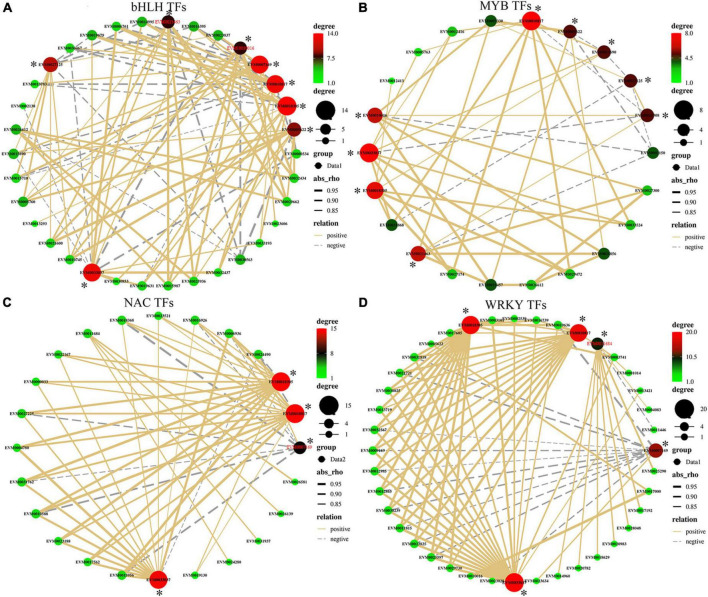
**(A**–**D)** Correlation of gene expression between the four main DEG transcription factor (TF) families (i.e., bHLH, WRKY, NAC, and MYB) and peroxidase activity-related genes. Line color represent positive (solid yellow) and negative correlation (dotted gray); line thickness represents the strength of the correlation; dot size/color represents the number of correlated objects. * represents the antioxidant genes.

Transcription factors can play a major role in regulating gene expression of target genes by binding to their promoter regulatory elements and thus affecting transcriptional activity of the targets. A total of 244 DEGs encoding TFs belonging to 20 major transcription factor families were identified across the three stages (Supplementary Table 5). Members of the bHLH (42/244), WRKY (40/244), NAC (30/244), and MYB families (21/244) represented the largest number of DEG transcription factors (Supplementary Table 5). To identify the potential functions of TFs that are associated with antioxidant enzyme-related genes, a correlation analysis of gene expressions between these four TF families and POD-related genes (POD, Supplementary Table 6) was performed (Figure 6 and Supplementary Table 7). *CmbHLH130 (EVM0032437)* was positively correlated with the expression of POD pathway genes *EVM0033037* (*r* = 0.98), *EVM0018305* (*r* = 0.95), and *EVM0010817* (*r* = 0.92), while *CmIRL3* (*EVM0030563*) was negatively correlated with the expression of *EVM0027125* (*r* = –0.86), *EVM0026612* (*r* = –0.82), *EVM0021463* (–0.91), *EVM0010016* (–0.93), and *EVM0005622* (–0.95). Expression of the POD gene *EVM0033037* positive correlated with 20 members of WRKY TFs family (Figure 6D and Supplementary Table 7) (20 out of 40), while the expression of POD genes *EVM0018305*, *EVM0011684*, and *EVM0010817* correlated with 19, 9, and 18 WRKY TFs, respectively, highlighting that WRKY TF members mostly positively correlated with these 4 POD-related genes. In contrast, only POD gene *EVM0007169* has shown a negative correlation with the expression of a large suite of WRKY family genes (12 out of 40). This trend was also apparent in the correlation network of NAC with POD, where *EVM0018305* and *EVM0010817* (both 13 out of 30) were positive correlated with NAC expression (Figure 6C and Supplementary Table 7). For the MYB family, the majority of members showed a positive correlation with POD-related gene expression (Figure 6B and Supplementary Table 8) with about 70% positive correlations. For example, *EVM0018305* only positively correlated with seven MYBs (7/21), while in contrast, *EVM0007169* only negatively correlated (5/21).

### Integrative Analysis of MAPK Signaling Pathway Genes Expression and Antioxidant Enzyme Activities

**FIGURE 7:**
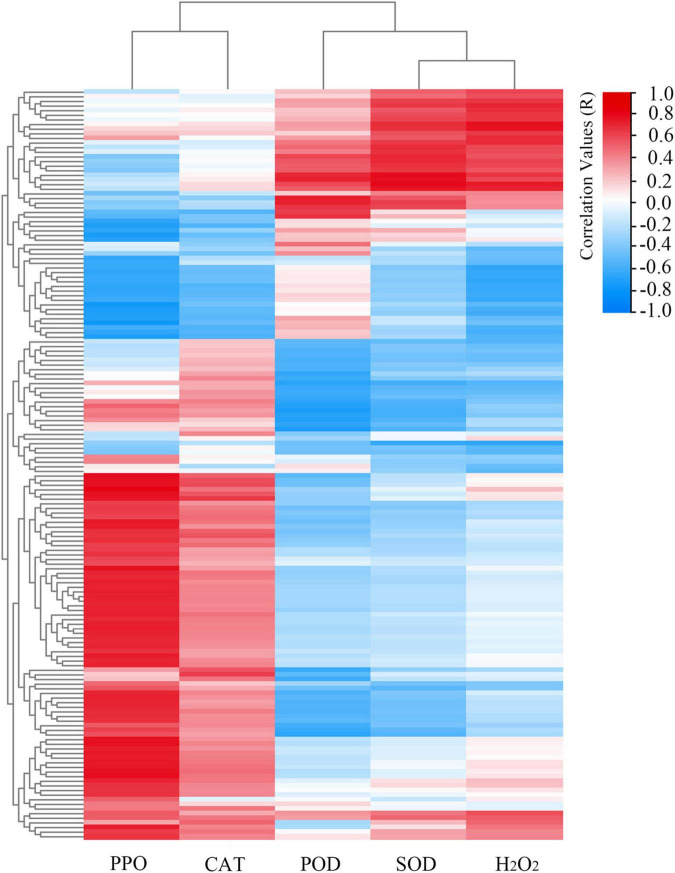
Representative candidate genes of MAPK signaling pathway likely linked to the H_2_O_2_ content and the main antioxidant enzyme activities (i.e., PPO, CAT, POD, and SOD). Scale bar represents the correlation values.

Based on the transcriptomics data and antioxidant enzyme activities data, we constructed a set of correlation networks between the five main antioxidant enzyme activities (i.e., PPO, CAT, POD, SOD, and H_2_O_2_) and the related MAPK signaling pathway gene expressions of 6 tested samples (stages A, B, and C of “HL” and “SW”). As shown in Figure 7 and Supplementary Table 8, most of MAPK signaling pathway members showed a positive correlation with PPO and CAT activities, while the majority was negatively associated with POD, SOD, and H_2_O_2_ activity/content. POD was significantly correlated with the MAPK-related genes basic leucine zipper 61 (*EVM0029813*, *r* = 0.84, *p*-Value < 0.05), receptor-like protein 12 (*EVM0032794*, *r* = 0.89, *p*-Value < 0.05), and the transcription factor MUTE (*EVM0020177*, *r* = 0.87, *p*-Value < 0.05). In contrast, PPO activity was positively correlated to several gene members of the MAPK signaling pathway.

### Correlations of Antioxidant Enzyme Activities and Expression of Antioxidant Enzyme Genes

To identify the antioxidant enzyme genes that may correlated to antioxidant enzyme activities, we constructed a set of correlation networks among the five main antioxidant enzyme activities (i.e., PPO, CAT, POD, SOD, and H_2_O_2_) to evaluate the expression of antioxidant enzyme-related genes. The results revealed that PPO activity was positively correlated with *EVM0026323* (*r* = 0.85, *p*-Value < 0.05) and *EVM0006016* (*r* = 0.72, *p*-Value < 0.05); CAT activity was positively correlated with *EVM002624729* (*r* = 0.81, *p*-Value < 0.05); POD activity was positively correlated with *EVM0026612* (*r* = 0.89, *p*-Value < 0.05), *EVM0005622* (*r* = 0.84, *p*-Value < 0.05), and negatively correlated with *EVM0011684* (*r* = –0.71, *p*-Value < 0.05). SOD activity was positively correlated with *EVM0032156* (*r* = 0.9, *p*-Value < 0.05), *EVM0033049* (*r* = 0.79, *p*-Value < 0.05), while *EVM0005687* expression was negatively correlated (*r* = –0.79, *p*-Value < 0.05) with buds H_2_O_2_ contents (Supplementary Table 9). All the aforementioned antioxidant enzyme activity-related genes could be important candidates for further Chinese chestnuts “antioxidant enzyme” study.

### Effect of Gall Wasp *Dryocosmus kuriphilus* Infestation on Expression of Terpene Synthase Enzymes

Terpenes are a class of secondary metabolite volatile compounds derived from the mevalonate and MEP substrate pathways that are often associated with direct and indirect defenses to pests and pathogens. In this study, GWDK feeding exhibits a significant effect on the elicited expression of terpene synthase (TPS) genes (Supplementary Table 10). For example, the expression of TPS gene *EVM0000042* was annotated as (*E,E*)-alpha-farnesene synthase and presented an 8-fold higher expression in HL than SW at the initiation stage A (Supplementary Table 10), while two (*3S,6E*)-nerolidol synthase genes *EVM0024276* and *EVM0000019* were induced 3- to 12-fold in both cultivars after infestation (Supplementary Table 10). Interestingly, (α)-farnesene and (*E*)-nerolidol have both been reported to be associated with herbivory defenses in plants (Jing et al., 2021).

### Validation of Differentially Expressed Genes by Quantitative Reverse Transcription-PCR

**FIGURE 8:**
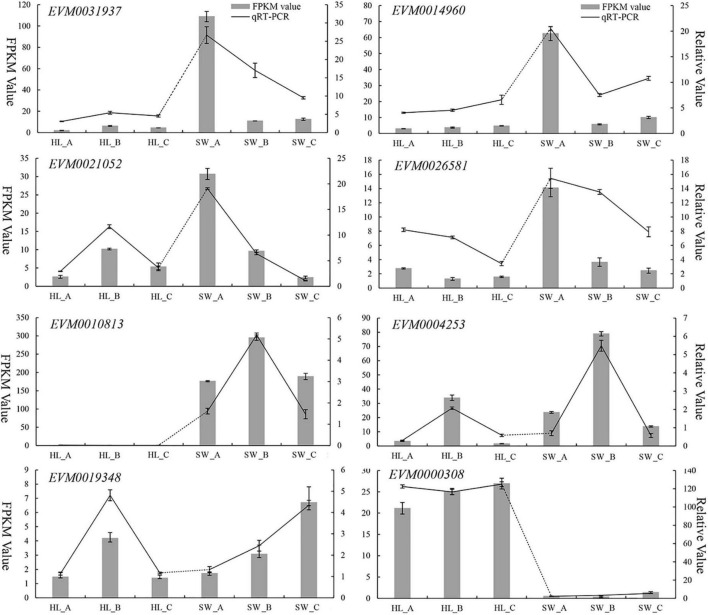
Quantitative reverse-transcriptase (qRT)-PCR validation of DEGs expression in developing “HL” and “SW” chestnut buds vs. by RNA-Seq. dark line: qRT-PCR expression, gray bars: transcriptomics RNA-Seq analysis [fragments per kilobase per million reads (FPKM)].

To validate the expression patterns for the DEGs generated by RNA-Seq, the expression levels of 8 randomly selected genes were further analyzed by qRT-PCR across the 6 sequence samples (i.e., 3 biological repeats per samples**).** The qRT-PCR data showed very similar patterns and trends of expression compared with the RNA-Seq transcriptomics data (Figure 8 lines vs. bar graphs). These results validate that the RNA-Seq data can be considered reliable and reproducible by an independent method (i.e., qRT-PCR) using a random selection of genes (*n* = 8).

## Discussion

The oriental gall wasp *D. kuriphilus* (GWDK) is considered the most serious pest to the Chinese chestnut industry, and infestation results in gall formation on vegetative parts such as buds, leaves, and petioles. It also contributes to large losses in fruit yield due to weakening of the plant health and indirectly by plants becoming more susceptible to other pests and diseases. The molecular and physiological aspects of GWDK infestation in European chestnuts (*Castanea sativa*, Fagaceae) have been previously examined in several studies (Sartor et al., 2012, 2015; Acquadro et al., 2020). The interaction of a specialist herbivore with a plant host can be exploited to dissect the architecture of plant defense signaling networks that can induce plant resistance or tolerance (Walling, 2008; Kaloshian and Walling, 2016). However, the molecular mechanisms and targets involved in GWDK infestations of Chinese chestnuts remain largely unknown. In this study, the comparative transcriptomic analysis of susceptible “HL” and the partially resistant “SW” Chinese chestnut (*C. mollisima*) buds at different stages during GWDK infestation were examined. A large number of DEGs that differed among the budburst stages of the 2 species were observed. Additionally, through KEGG enrichment analysis, a large portion of the DEGs were involved in the likely recognition of specific elicitors and herbivore damage, and substantial variations were identified in four of the main pathways that were enriched, including plant hormone signal transduction, MAPK signaling, ribosome biogenesis in eukaryotes, and the peroxisome pathway.

Oxidative stress signaling is proposed to be an important process induced by herbivory infestation (Caverzan et al., 2016), where H_2_O_2_ and ROS are common elicitors of defense signaling that are involved in the elicitation of plant responses to herbivory attack. Oxidative enzymes have been reported as a signaling factor to increase the expression of genes related to biotic stresses (Maffei et al., 2007). This study highlights that POD, H_2_O_2_, and SOD were higher in resistant cultivar leaves compared with susceptible HL at most of the development stages of infestation, while CAT and PPO activities were mostly higher in susceptible (i.e., HL) leaves (Figure 2). Through the GO and KEGG enrichment analysis, we demonstrated that the “catalytic activity” was a significant GO-enriched term in stages B and C, which relates to enzyme catalysis (Figure 5), while in the KEGG enrichment analysis, “MAPK signaling pathway” and “plant hormone signal transduction” featured prominently in all three stages (Table 2). Modifying the levels of SOD, POD, and CAT gene expression and enzyme activities affects the H_2_O_2_ balance through production and scavenging and may directly (insecticidal) or indirectly (defense signaling) influence galls formation and growth. Our results were consistent with the studies carried out on tea (Ning et al., 1996) and wheat (Han et al., 2009) plants that stronger activities of glutamine dehydrogenase and PAL were observed in the shoots, which were resistant to pink mite feeding. In the wheat study, resistance against cereal aphid was associated with higher constitutive PAL and PPO activity, while aphid infestation further enhanced the levels of PAL and PPO activities in both resistant and susceptible cultivars. In contrast, aphid infestation induced POD activity in both tested cultivars, especially in susceptible ones. Therefore, further investigation into the complex relationship between several defense pathways in relation to GWDK infestation and the role of the specialized antioxidant enzyme systems is required.

Constitutive defense mostly directly acts on the invading agent, while induced responses such as ROS and phytoalexins defense signals, in addition to direct defense, can benefit the plant by employment “on demand.” For example, herbivore-induced release of volatile compounds has received much attentions, including terpenoids, aromatic compounds, and green leaf volatiles, and transcriptional analysis of these responses has been widely studied (He et al., 2020a; Pingault et al., 2021). Considering that a number of TPS genes were significantly upregulated after GWDK infestation in both HL and SW plants at the later infestations stages B and C compared with the initially budburst stage A, we hypothesized that these TPS genes are good candidate genes for further study that may contribute to the production and emission of several terpene compounds either involved in inhibition of herbivory or by attracting predatory insects (Clancy et al., 2020). Our study provides some indirect evidence to support a network between herbivory feeding and terpene production and release, but more studies are required to link this to the terpenoid secondary metabolites that are induced.

In the process of herbivory feedings, transcription factors often play pivotal roles in regulating transcriptional responses by the plant. The functional category of “transcription factors” was significantly enriched in the transcript expression profiles of many biotic defense-related transcriptomics comparative studies, and the WRKY TF class is widely reported in the context of biotic and abiotic stress responses (Bai et al., 2018). Induction of the expression of WRKY TF genes in response to herbivory was observed in *N. attenuata* (Skibbe et al., 2008), rice (Akagi et al., 2014), and other plants (Kundu and Vadassery, 2021), which affected the biosynthesis of defensive secondary metabolites or by reprogramming the expression of associated pathway genes. Apart from WRKY TFs, other classes of TFs associated with herbivory infestations have also been widely reported, for example, in rice, leaf, and phloem feeding by brown planthopper (BPH) induced the transcript levels of genes encoding transcription factors of the AP2/ERF, MYB, bZIP, and bHLH families (Li et al., 2021). *OsMYB30* may play a key role in modulating the resistance of rice to BPH by regulating the biosynthesis of salicylic acid and expressions of genes in the PAL pathway (He et al., 2020b). In Chinese chestnuts, the genetic mechanisms of TF action involved in GWDK feedings have not been previously experimentally elucidated. We identified 244 TF members belonging to 20 major TFs families that were differentially expressed in the susceptible vs. resistant cultivar during one or more of the stages of bud infestation by GWDK. Among them, four transcription factors family, namely, bHLH, WRKY, NAC, and MYB, were widely represented (Supplementary Table 5). Candidate TFs identified in this study can contribute in future to a more detailed understanding of how the transcriptional regulatory networks may contribute to a successful herbivory resistance response.

When attacked by herbivories, plants can respond in terms of signal transduction *via* phytohormonal pathways, induce gene expression changes, which may lead to the biosynthesis of secondary metabolites and defense proteins (Erb and Reymond, 2019). Jasmonic acid (JA) has been long recognized as a pivotal mediator that regulates a myriad of plant developmental and biotic stress responses especially against herbivores, and pathogens as well as abiotic stress responses such as wounding and UV damage (Sun et al., 2011; Alhaithloul and Soliman, 2021). Chewing herbivores such as caterpillars and piercing-sucking insects such as white flies can significantly induce the jasmonate pathway in *Arabidopsis thaliana* (Zhang et al., 2013, 2020). Methyl jasmonate treatment can also induce the defense responses in bilberry (Benevenuto et al., 2019), Norway spruce (Mageroy et al., 2020), and Jacobaea vulgaris (Wei et al., 2021). In our study, KEGG enrichment identified “plant hormone signal transduction” and “MAPK signaling” (Supplementary Table 3) among the top enriched pathways in all three stages. Additionally, five proteins annotated as TIFY protein and JA-related transcription factors MYC2 were detected and showed higher expression in resistant SW compared with the susceptible HL variety.

Understanding of insect-plant interactions is of interest not only from an ecological and evolutionary perspective but also for the development of novel crop protection strategies such as through resistance breeding. The data presented in this study provide genetic information for the discovery of new regulatory steps (TFs) and can identify novel biosynthetic genes such as terpene synthases that may, for example, produce repellent, antifeeding, or insecticidal compounds or act as volatile signals that attract natural enemies of herbivores. WRKY TFs and related genes may influence the levels of target modifying/sequestration enzymes (e.g., PAL and PPO) involved in the GWDK resistance. Our correlation analysis of TFs and oxidative enzyme levels (Figure 6 and Supplementary Table 7) revealed that aforementioned enzymes and TFs could potentially contribute to the resistance process as part of a network of induced defense responses.

**FIGURE 9:**
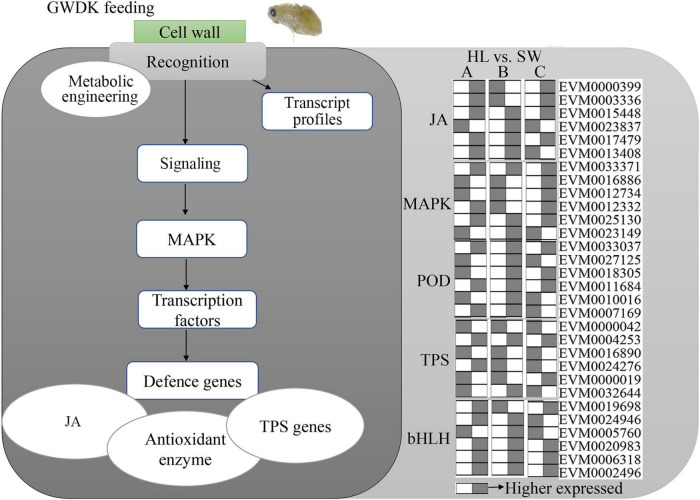
Genetic model of Chinese chestnut buds responses upon GWDK infestation. The heatmap includes the top 6 highest expressed genes in jasmonic acid (JA), MAPK, POD, terpene synthase (TPS), and bHLH gene classes; gray color represents higher expressed of a certain gene compared with white (lower).

To further categorize and visualize the various functions and components of the GWDK-responsive genes in buds, all DEGs were assembled into a model for GWDK responses (Figure 9). A large proportion of the DEGs belong to the signals derived from JA and MAPK signaling and involves enzymatic metabolism such as POD and TPS. Most of these pathways have been reported to play major roles in the stress responses in plants.

## Conclusion

In summary, we investigated the defense response of GWDK-susceptible and GWDK-partial resistant Chinese chestnuts through *de novo* transcriptome sequencing and oxidative enzyme metabolism analysis. It shows that the activities of CAT and POD, PPO, and SOD were closely associated with the Chinese chestnuts GWDK infestation, where the CAT and PPO activities were significant higher in HL buds compared with SW. RNA-Seq transcriptomic analyses of HL and SW leaves revealed that various metabolic pathways involved in GWDK biotic stress/defense responses, such as plant hormone signal transduction, MAPK signaling, ribosome biogenesis in eukaryotes, and peroxisome pathway, were enriched. Moreover, changes of gene expression levels of terpenoid biosynthetic, plant hormone signaling transduction, and WRKY TFs were observed. Further study on the herbivory feeding and related DEGs can provide us more detailed insights into GWDK resistance mechanisms, and this knowledge can be exploited and used in agriculture to inform better management practices and in plant breeding to improve the genetics and ultimately the quality and quantity of Chinese chestnuts production.

## Data Availability Statement

Publicly available datasets were analyzed in this study. This data can be found here: https://www.ncbi.nlm.nih.gov/sra, PRJNA791965.

## Author Contributions

CZ, WW, and YC participated in the design of the study and revised the manuscript. WW, SZ, and YZ contributed to performing the experiments, data analyses, and manuscript writing. CZ conducted antioxidant enzyme metabolite data analyses. MK-U-R and NN contributed to the writing and reviewing of the manuscript. All authors contributed to the article and approved the submitted version.

## Conflict of Interest

NN was employed by the New Zealand Institute for Plant and Food Research Ltd. The remaining authors declare that the research was conducted in the absence of any commercial or financial relationships that could be construed as a potential conflict of interest.

## Publisher’s Note

All claims expressed in this article are solely those of the authors and do not necessarily represent those of their affiliated organizations, or those of the publisher, the editors and the reviewers. Any product that may be evaluated in this article, or claim that may be made by its manufacturer, is not guaranteed or endorsed by the publisher.

## Footnotes

1
https://ngdc.cncb.ac.cn/search/?dbId=gwhandq=GWHANWH00000000

2
http://www.geneontology.org/

3
https://www.kegg.jp/

4
https://www.hardwoodgenomics.org/

5
https://www.omicstudio.cn/tool
